# Active macromolecules of honey form colloidal particles essential for honey antibacterial activity and hydrogen peroxide production

**DOI:** 10.1038/s41598-017-08072-0

**Published:** 2017-08-09

**Authors:** Katrina Brudzynski, Danielle Miotto, Linda Kim, Calvin Sjaarda, Liset Maldonado-Alvarez, Henryk Fukś

**Affiliations:** 1 Department of Biological Sciences, Brock University, 1812 Sir Isaac Brock Way, St. Catharines, Ontario Canada; 2 Department of Mathematics & Statistics, Brock University, 1812 Sir Isaac Brock Way, St. Catharines, Ontario Canada

## Abstract

Little is known about the global structure of honey and the arrangement of its main macromolecules. We hypothesized that the conditions in ripened honeys resemble macromolecular crowding in the cell and affect the concentration, reactivity, and conformation of honey macromolecules. Combined results from UV spectroscopy, DLS and SEM showed that the concentration of macromolecules was a determining factor in honey structure. The UV spectral scans in 200–400 nm visualized and allowed quantification of UV-absorbing compounds in the following order: dark > medium > light honeys (p < 0.0001). The high concentration of macromolecules promoted their self-assembly to micron-size superstructures, visible in SEM as two-phase system consisting of dense globules distributed in sugar solution. These particles showed increased conformational stability upon dilution. At the threshold concentration, the system underwent phase transition with concomitant fragmentation of large micron-size particles to nanoparticles in hierarchical order. Honey two-phase conformation was an essential requirement for antibacterial activity and hydrogen peroxide production. These activities disappeared beyond the phase transition point. The realization that active macromolecules of honey are arranged into compact, stable multicomponent assemblies with colloidal properties reframes our view on global structure of honey and emerges as a key property to be considered in investigating its biological activity.

## Introduction

Honey chemical composition is a complex mixture of structurally and functionally diverse molecules that originate from plant nectars, pollen and from honeybee itself. Nectars provide honey with water, sugars (such as sucrose and hexoses), free amino acids (such as proline and phenylalanine), polyphenols, volatile organic compounds, alkaloids and minerals. Pollen grains supply honey with vitamins, lipids including fatty acids, phytosterols, caroteinoids, pollen specific proteins, amino acids and minerals. After collection, nectars become transformed to honey after mixing with glandular secretion of honeybee. The secretion of hypopharyngeal glands is rich in enzymes involved in carbohydrate metabolism such as amylases, invertases, glucosidases and glucose oxidases^1^. The hypopharyngeal glands also provide honey with proteins of royal jelly, such as members of Major Royal Jelly Proteins (MRJPs) family that comprise the most abundant group of proteins in honey^2^. In addition, they also enrich honey with monosaccharides, fatty acids and vitamins B, C and E^2, 3^.

After placing droplets of the nectar-pollen mixture in open comb cells, enzymes of bee hypopharyngeal glands start to hydrolyze nectar’s carbohydrates to glucose and fructose Subsequently, through nectar evaporation by the bee’s accelerated wing fanning, these sugars are concentrate to about 82%. In this process, up to 70% of water is removed from honey, generating the supersaturated sugar solution with considerably restricted amounts of free water. As a result, honey compounds become confined to extremely crowded milieu that enhances nonspecific, non-covalent interactions between molecules.

The conditions in ripened honey can be compared to the macromolecular crowding inside the cell^4–6^. High concentrations of macromolecules such as DNA, RNA or proteins that occupy considerable part of the cytosol volume can effectively reduce the volume of water available for other molecules. This excluded volume effect is responsible for enhancing attractive forces between molecules by decreasing the dissociation rates and molecular mobility^6–8^. With reference to honey, its compositional analysis revealed that high molecular weight components such as proteins, multi-subunits enzymes, polyphenols, polymerized Maillard reaction products, comprise only a percentage of total mass. However, from the perspective of macromolecular crowding, we hypothesized that the effective concentration of these macromolecules would increase dramatically in the presence of crowding molecules, due to excluded volume effect. Enhanced association reaction between proteins induced by crowding has been observed in the presence of artificial crowders such as BSA^9^ or dextran, Ficoll and PEG^10^ that was used to mimic the intracellular environment. In contrast, we considered a supersaturated concentration of fructose and glucose as crowding molecules that confine and immobilize honey macromolecules, enforcing their intermolecular interactions and the formation of larger structures. These events would explain our previous data on the formation of melanoidins^11^ and high molecular protein-polyphenol complexes in honeys^12^.

It is our prediction, that upon honey dilution, the physical forces underlying the intermolecular interactions (polar, hydrogen-bond and hydrophobic interactions) will be changed due to a destabilizing effect of water molecules and the reduced sugar concentration. Thus, the main aim of this study was to analyze whether macromolecular crowding in ripened honeys affects honey conformation differently than in dilute, aqueous solution and whether these conformational changes have a relevance to honey antibacterial activity and hydrogen peroxide production.

We also explored whether the method of UV spectral profiling would capture the most effective/descriptive features of honey structural changes in response to dilution.

## Results

### Macromolecular crowding conditions in honey

The supersaturated concentration of sugars in honey creates conditions resembling macromolecular crowding in the cell. The high concentration of fructose and glucose in honey reduces water available for other molecules, leading to increased concentration of solutes. Therefore, we measured water activity (aw), total sugar concentration in °Brix, moisture content and specific gravity (SG) as physical parameters that could define molecular crowding in honey (Table 1). There were no significant differences between tested honeys with regards to these parameters (Table 1). Water activity ranged between 0.535 and 0.608 with the average of 0.5762 ± 0.005, Brix values ranged from 79.9 to 84.2 with the average of 81.85 ± 0.29, moisture content ranged from 14.4 to 20.2 with the average of 16.69 ± 0.31. The high content of sugars and relatively low water activity indicate that most of water was bound to sugars. Since the total sugar concentrations, measured in °Brix was similar, the conformational constraints that sugars exert on macromolecules were essentially of the same magnitude in all tested honeys. It is therefore assumed that a variable influencing honey global structure would relate mainly to the concentration of macromolecules in different honeys.

**Table 1:** Water activity, refractive index, percentage of moisture and sugars and specific gravity of tested honeys.

	Honey	Source	Water activity (aw)	Refractive index	Moisture (%)	Brix	Specific gravity (SG)
*Light*	H210	wildflower	0.535	1.4964	16.4	82.4	1.4295
	H62	borage	0.598	1.4967	16.0	82.6	1.4310
	H87	wild clover	0.535	1.4948	16.8	81.8	1.4254
	H63	firewood	0.526	1.4902	18.6	79.9	1.4129
	68	clover/canola	0.574	1.4864	20.2	78.2	1.402
	H64	dandelion	0.563	1.4915	18.0	80.4	1.4171
	H11	clover bland	0.550	1.4952	16.4	82.0	1.4282
	H66	blackberry	0.608	1.4975	15.6	82.9	1.4338
*Medium*	H20	clover/buckwheat	0.594	1.4910	18.2	80.2	1.4156
	H96	buckwheat	0.575	1.4965	16.0	82.5	1.431
	H207	canola/willow	0.574	1.4965	16.0	82.5	1.431
	H99	sunflower	0.573	1.4923	17.8	80.7	1.419
	H224	buckwheat	na	1.4964	16.2	82.3	1.4295
	H225	wildflower	na	1.496	16.4	82.4	1.4295
	H223	buckwheat	na	1.4923	17.8	80.7	1.419
	H208	buckwheat	0.580	1.4900	18.6	79.8	1.4129
	H221	buckwheat	0.575	1.4946	17.0	81.8	1.4257
*Dark*	H76	buckwheat	0.591	1.4975	15.6	82.9	1.4338
	H226	buckwheat	0.601	1.5000	14.4	84.2	1.4428
	H58	buckwheat	0.576	1.4910	18.2	80.2	1.4156
	H77	buckwheat	0.597	1.4964	16.2	82.3	1.4295
	H23	buckwheat	0.594	1.4965	16.0	82.5	1.431
	H220	buckwheat	0.601	1.4988	15.8	83.4	1.4370
	H149	buckwheat	0.601	1.5000	14.4	84.2	1.4428
	H125	buckwheat	0.589	1.5000	15.0	83.5	1.4381

### UV spectral profiling is a rapid method to differentiate between honeys

UV spectral profiling was applied to quantify honey’s compounds such as proteins, polyphenols and Maillard reaction products. Proteins, peptides and free amino acids have absorbance maxima in the 200–230 nm and 250–290 nm range, which overlap with the absorption of hydroxybenzoate and hydrocinnamate class of phenolic acids that absorb in the 200 to 290 nm and at 270 to 360 nm ranges, respectively^13–15^. The early stage Maillard reaction products show a broad absorption range from 220 nm to 350 nm^16^. The oligo- and polysaccharides have absorbance maxima at 230 nm, while honey monosaccharides, glucose and fructose, absorb UV light at 180 to 200 nm and contribute little to the UV profiles.

**Figure 1:**
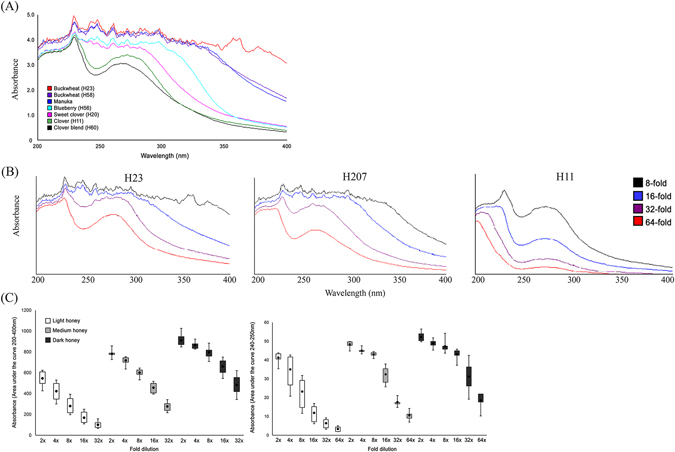
UV extinction spectra at 200 to 400 nm range of different honeys at 8-fold dilution (**A**), changes in the shape and magnitude of spectral profiles of dark (H23, buckwheat), medium (H207, canola/willow blend) and light (H11, clover) honeys upon 8 to 64-fold dilution (**B**), the non-linear rate of decrease of the AUC 200–400 nm and AUC 240–250 nm in medium (grey boxes) and dark honeys (black boxes) as oppose to in light honeys (open boxes) upon dilution.

Initially, we used UV spectral profiling for a sole purpose to distinguish honey varieties based on how strongly they absorb UV light in 200–400 nm range. Overlay of collected absorption scans of different honeys provided a rapid, visual display of the significant differences in the total concentration of UV absorbing compounds between light, medium and dark honeys (Fig. 1A). Each honey color group gave rise to a characteristic UV spectral shape and magnitude. Due to high concentration of UV absorbing compounds, UV spectra of medium and dark honeys appeared as a complex, elaborate, poorly resolved absorption curve of overlapping peaks. The most characteristic feature of the elaborate spectral profile in these honeys was the presence of double absorption peaks at 240–250 nm wavelengths. In contrast, light-color honeys, such as blueberry or clover produced mostly two-peak UV spectra demonstrating much fewer number of UV absorbing compounds (Fig. 1A). Although the nature of organic molecules responsible for 240–250 nm peaks remains unclear, the concentration of compounds with large conjugated systems of double bonds such as polyphenols might influence peak wavelengths and absorption intensities in these regions^14^. The large conjugated systems also absorb visible light and appear as a color. The color of medium and dark honeys (measured at A560 nm) supports the presence at increased levels of the compounds with the large conjugated system in these honeys as compared to light honeys.

### UV spectral profiles depend on the concentration of UV absorbing compounds and predict honeys conformational stability upon dilution

The interpretation of UV spectra of honeys posed a challenge because of the high concentration of UV absorbing compounds. To reduce spectrum complexity and absorbance intensity, UV spectroscopy was preceded by diluting honey samples from 2-fold to 64-fold with Mili-Q water. Overlay of UV spectra of medium and dark honeys, taken at consecutive two-fold dilutions, showed a strikingly slow reduction of UV absorbance up to 16-fold dilution and also a slow reduction in complexity of the spectral profiles (Fig. 1B). In contrast, the intensity of UV spectra of light honeys was sensitive to water dilution and showed a gradual decrease over the entire dilution range (Fig. 1B). Thus, high concentrations of UV absorbing compounds in medium and dark honeys provided increased conformational stability against dilution with water.

### Changes in UV spectral profiles upon honey dilutions deviate from linearity in medium and dark honeys

The inability to reduce UV absorbance of honeys by dilution with water to the manageable level below A=2.0 AU under our experimental conditions suggested that the observed high absorptivity was most likely caused by light scattering. The obtained spectra were therefore the extinction spectra representing both absorption and light scattering. To carry out investigation on conformational stability of honeys upon dilution, we needed a method to estimate the concentration of UV absorbing compounds in each honey. There are no easy techniques to separate absorbance from light scattering without sample processing. To our advantage, the observed changes in the extinction spectrum between different honeys upon dilution indicated that absorption and light scattering were intrinsic property of each honey and were changing in synergistic way (Table 2, Fig. 1C). Therefore, we quantified UV absorbing compounds by measuring the integrated area under the curve (AUC) of scanned honey samples collected at two-fold dilutions using the SWIFT II waves can software (Ultrospec 3100 Pro UV/Vis scanning spectrophotometer), knowing that the AUC represents both absorbance and light scattering. The total concentration of UV absorbing compounds, expressed as the value of AUC (200 nm–400 nm) and AUC 240–250 nm were plotted as a function of honey dilution (Table 2).

**Table 2:** Honey color and concentration of UV absorbing compounds, measured as AUC 200–400 nm and AUC 240–250 nm, versus honey dilution.

	Honey	Color	AUC					AUC					
		A560–720 nm	200–400 nm					240–250 nm					
			2x	4x	8x	16x	32x	2x	4x	8x	16x	32x	64x
*Light*	H210	0.03	597.6	482.0	347.2	221.1	114.6	39.6	38.6	28.3	15.1	8.1	5.1
	H62	0.05	466.4	348.4	217.9	125.2	78.7	40.9	26.6	13.7	6.6	3.7	2.0
	H87	0.05	506.9	341.9	210.0	108.9	66.4	43.4	40.9	26.6	13.6	6.6	3.5
	H63	0.06	426.3	300.2	196.8	116.4	64.7	35.4	20.8	11.6	6.1	3.2	1.9
	H68	0.10	520.0	377.0	252.0	146.7	88.2	41.6	26.8	15.1	7.4	4.0	2.0
	H64	0.12	624.2	529.3	393.1	249.8	157.8	43.3	42.3	31.8	16.9	9.2	4.5
	H11	0.13	621.0	492.0	262.0	153.0	93.0	43.8	42.7	31.8	15.6	8.9	4.3
	H66	0.14	604.2	512.2	364.1	224.5	138.2	42.7	41.4	27.0	13.8	7.4	3.5
*Medium*	H20	0.24	726.4	635.6	531.6	396.3	216.0	44.8	43.4	40.8	28.2	15.9	7.0
	H96	0.25	858.0	728.6	584.5	406.2	219.9	48.8	47.6	43.9	26.8	16.7	9.1
	H207	0.25	777.6	691.8	578.3	440.0	237.4	46.0	45.0	42.6	35.4	17.2	11.2
	H99	0.26	761.9	743.4	598.3	394.6	256.9	47.9	46.5	43.9	25.7	14.8	8.5
	H224	0.27	788.8	722.0	615.8	497.7	277.4	47.9	44.5	42.6	34.8	17.3	10.8
	H225	0.28	775.2	701.1	588.8	456.7	298.7	46.9	43.3	41.7	32.0	17.4	11.0
	H223	0.33	776.0	753.8	626.4	483.4	302.7	45.2	44.7	43.2	33.2	17.3	10.5
	H208	0.37	784.8	741.5	641.5	517.9	336.6	47.8	45.1	44.5	37.3	20.5	14.2
	H221	0.38	787.8	751.1	644.8	515.9	341.8	46.7	44.8	43.6	37.9	21.1	12.0
*Dark*	H76	0.61	886.0	869.7	759.2	608.4	388.5	50.2	48.4	47.0	42.7	27.0	17.9
	H226	0.62	848.0	826.6	708.0	587.7	429.1	46.7	45.3	43.7	42.0	28.0	17.0
	H58	0.99	857.6	828.6	811.5	681.0	518.2	50.0	47.2	46.7	43.8	40.0	21.9
	H77	1.27	1026.0	922.6	883.7	745.0	553.0	50.6	51.3	54.2	45.8	34.0	25.0
	H23	1.53	941.8	872.4	807.7	703.4	558.0	56.5	51.8	47.3	44.8	35.1	17.9
	H220	1.57	875.0	856.2	832.7	749.4	619.9	53.8	49.4	45.8	45.3	42.4	22.0
	H149	1.58	958.0	891.0	805.8	651.0	441.0	49.4	48.2	47.7	42.8	23.8	19.0
	H125	1.60	892.0	836.0	760.0	545.3	343.0	50.4	49.0	45.8	37.2	19.2	7.8

We found that the obtained values of AUC 200–400 nm and AUC 240–250 differ significantly among light, medium and dark honeys (ANOVA, p < 0.0001) (Fig. 1C and Table 2). Tukey-Kramer Multiple Comparisons post hoc test confirmed that light honeys contained significantly lower concentrations of UV absorbing compounds compared to that of medium-color and dark-color honeys (p < 0.001) while medium group contained lower concentrations of UV absorbing compounds that that of dark honeys (p < 0.001) (Fig. 1C).

Next, we found that the rate of decrease in AUCs as function of dilution was non-linear in medium and dark honeys from 2- to 8-fold and 2- to 16-fold dilution, respectively.

Similarly, the rate of decrease in AUC 240–250 nm in medium and dark honeys was significantly slower and non-linear over 2- to 8-fold dilution range. In contrast, the rate of decrease in AUCs absorbing of light honeys was approximately linear with each dilution (Fig. 1C).

### Honey exists in two conformational states and undergoes a concentration-dependent phase transition

The non-linear decrease in absorbance of medium and dark honeys upon dilution was observed up to 8-fold and 16-fold for medium and dark honeys, respectively. After this point of dilution, the AUC 240–250 nm decreased abruptly and became linear with further dilution (Fig. 1C). This transition point changed the shape of UV spectra from elaborate to two-peak spectral profiles, similar to that of light honeys (Fig. 1A and B, Table 2).

We found that the value of AUC 240–250 nm higher than ≥40 mAU represented the threshold concentration needed to preserve the stable honey conformation, reflected by the elaborate UV spectra (Fig. 1C). The point of dilution that caused an abrupt decrease of the AUC 240–250 nm below ≤40 mAU value was referred to as the phase transition point.

The phase transition point separated two qualitatively different conformational states of honey: one, stable, water-resistant conformation that was induced by the high concentration of UV absorbing compounds, and another one, unstable, water- sensitive conformation that resulted from the low concentration of UV absorbing compounds in dilute solutions.

### Dynamic light scattering revealed a dynamic self-assembly and disassembly of honey macromolecules

UV spectra of medium and dark honeys showed very high absorptivity suggesting that a part of it is caused by light scattering. We therefore employed dynamic light scattering to analyze the size distribution profiles of macromolecular particles in three color groups; in medium (H207) and dark honeys (H220) that are characterized by high AUC values and light honey (212) of low AUC values (Table 2). Since insoluble particles and large aggregates preclude accurate DLS measurements, honeys were purified through a set of filtration steps (Materials and Methods) and then serially, two-fold diluted with Mili-Q water. Each honey dilution was subjected to DLS.

**Figure 2:**
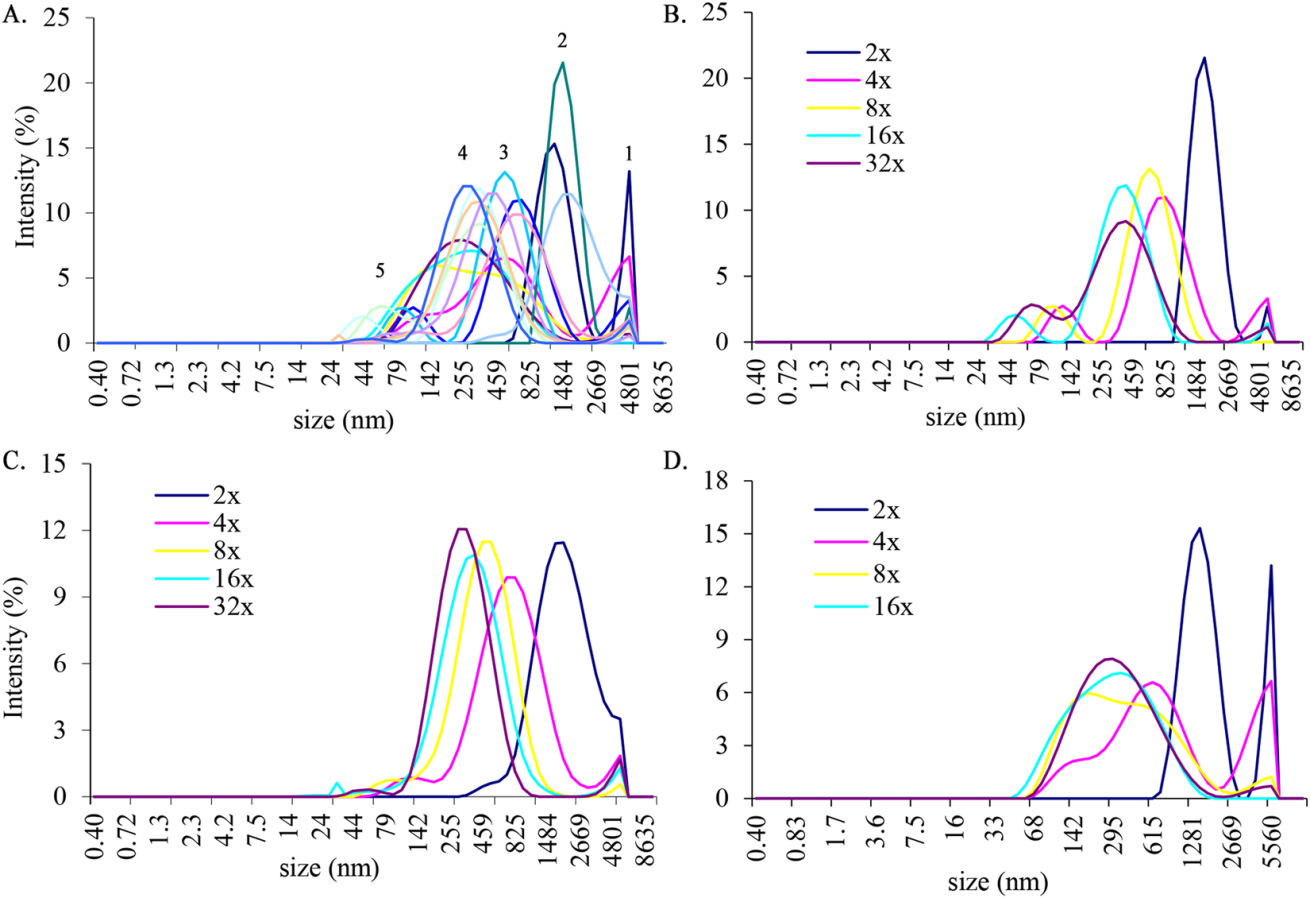
The particle size distribution by intensity in all honeys as a function of dilution in 2-fold to 32-fold range (**A**). The distribution of particle sizes in medium (H207) (**B**), light (H212) (**C**) and dark honey (H220) honey (**D**) as a function of dilution.

By overlaying graphs for dark, medium and light honeys, we obtained a complete view of particle size distribution (PSD) by intensity as a function of honey dilution (Fig. 2A). The range of intensity contributions from particles of similar diameters allowed their separation into five peaks. The particle populations in peaks 1, 2 and 5 differed significantly in a mean particle size, (p < 0.0001) (Fig. 2A). No significant difference was found, however, in the mean size diameter between peaks 3 and 4 (541.37 ± 73.13 nm and 259.85 ± 35.13 nm, respectively) (Fig. 2). These particle populations were common for all three honeys.

In fact, the individual graphs for the intensity–based PSD in dark, medium and light honeys suggested that honey dilutions led to the fragmentation of the honey micron-size superstructures into smaller particles in a quite orderly manner (Fig. 2B,C and D). Only in dark honey, H220, that had the highest concentration of UV absorbing compounds, the PSD was bimodal with the mean particle size of 5179.5 ± 379.5nm and 1308.6 ± 135.07 nm (peak 1 and 2 at 2-fold dilution) with intensity contribution of 13.2% and 15.3%, respectively (Fig. 2D). The peak intensity at 5179 nm decreased markedly in medium honey H207 and light honey H212 to 2.6% and 3.5%, respectively (Fig. 2B and C) and shifted heavily toward the particle size of 1718 nm in honey H207 and 1990 and 1718 nm in honey H212 with intensity 21.5% and 11.4%, respectively (Fig. 2B and C). Furthermore, diluting honey 4-fold led to degradation of micron size particles to nanoparticles of similar sizes (H207 and H212) with similar intensities of 10%. At this dilution honey H220 still retained the large particles of 5179 nm size as well as the fragments of 615nm of the same intensity of 6.5% (Fig. 2D). From that point of dilution, each subsequent two-fold dilution further reduced honey particle size (Fig. 2). Thus, honey dilution led to dissociation/degradation of large, micron- size particles to smaller size particles.

**Figure 3:**
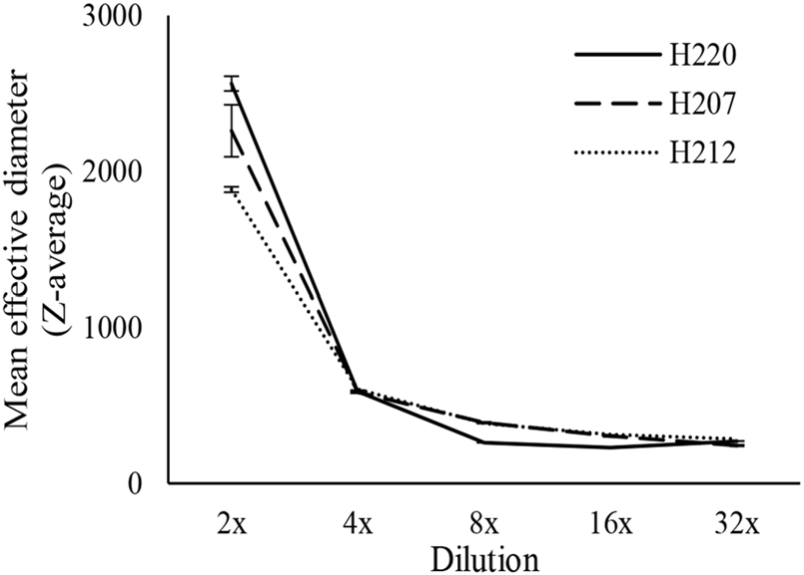
The change in a mean hydrodynamic diameter (Z-average) for dark, buckwheat honey (H220), medium-color canola/willow mixed honey (H207) and light, wildflower honey (H212) as a function of dilution.

When the mean effective diameters of the particles (Z-average) were measured as a function of honey dilution, the size of particles of buckwheat honey H220 have significantly larger diameters at 2-fold dilution than that of medium (H207) and light (H212) honeys with lower concentrations of UV absorbing compounds (Fig. 3). With further dilution, the rate of decrease in the effective diameter of particles became essentially superimposable, showing that molecular crowding is needed for the formation of macromolecular superstructures (Fig. 3). These results indicate that the size of honey particles depends on both, the high concentration of crowding molecules and the high concentration of macromolecules. High concentration of crowding sugars exerted the pressure on macromolecules to bring them closer together by reducing the available water, while the concentration of macromolecules was required to build micron-size, stable particles.

### SEM images support two-phase honey conformation

**Figure 4:**
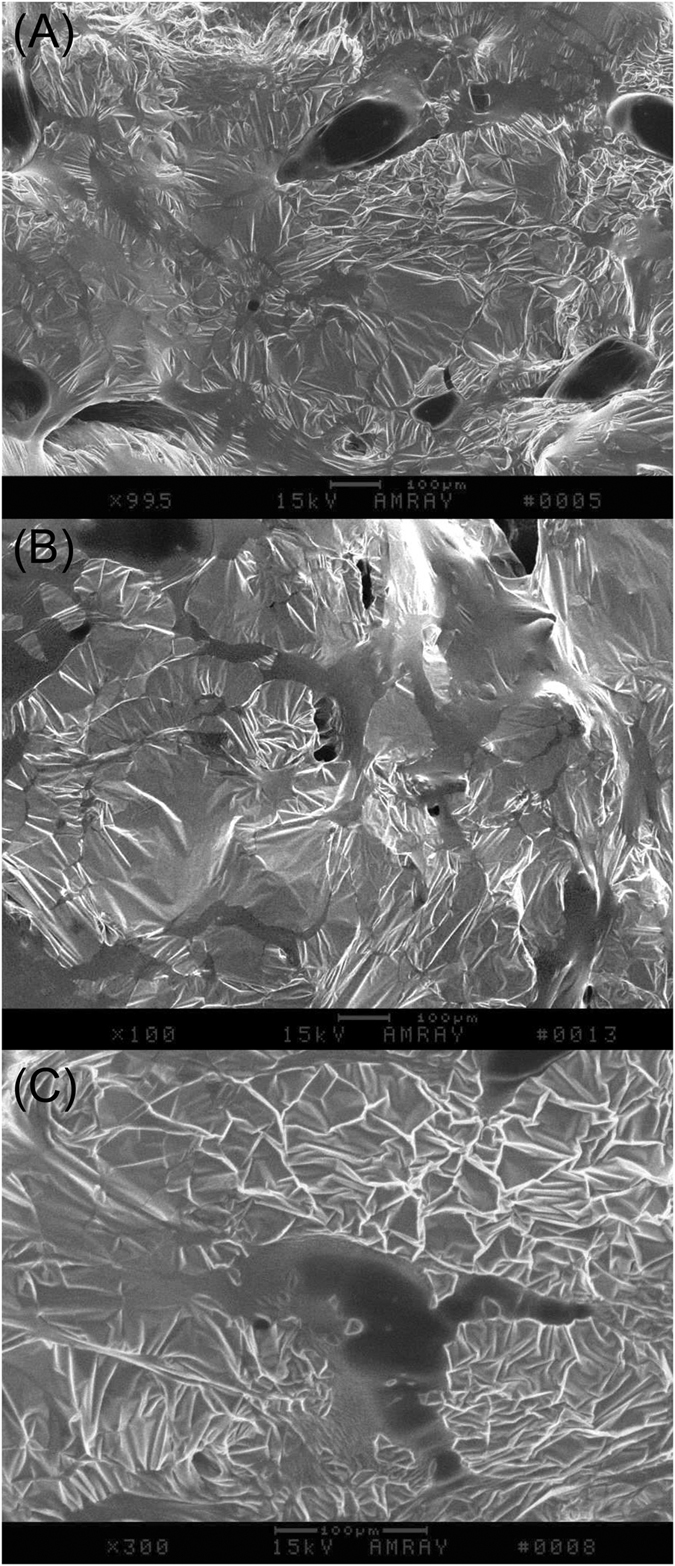
Representative SEM images of dark buckwheat honey H220 (**A**) and medium-color, H207 (**B**) honeys, indicating two-phase systems consisting of the dence globular structures dispersed in sugar solution. The scale bar is 100 µm, magnification of images (**A**) and (**B**) is 99.5x. (**C**) Micrograph of globule at the magnification of 300x. The scale bar is 100 µm.

Figure 4 shows SEM images of particle sizes formed in light, medium and dark honeys. The picture shows a two-phase system, formed under macromolecular crowding consisting of dense, spheroidal structures of varying sizes that are confined into spatially distinct areas in sugar solution.

The higher-power image of the spherical structure showed that its components interact with the surrounding milieu.

### Two-piece linear function model allows prediction of a phase transition point

**Figure 5:**
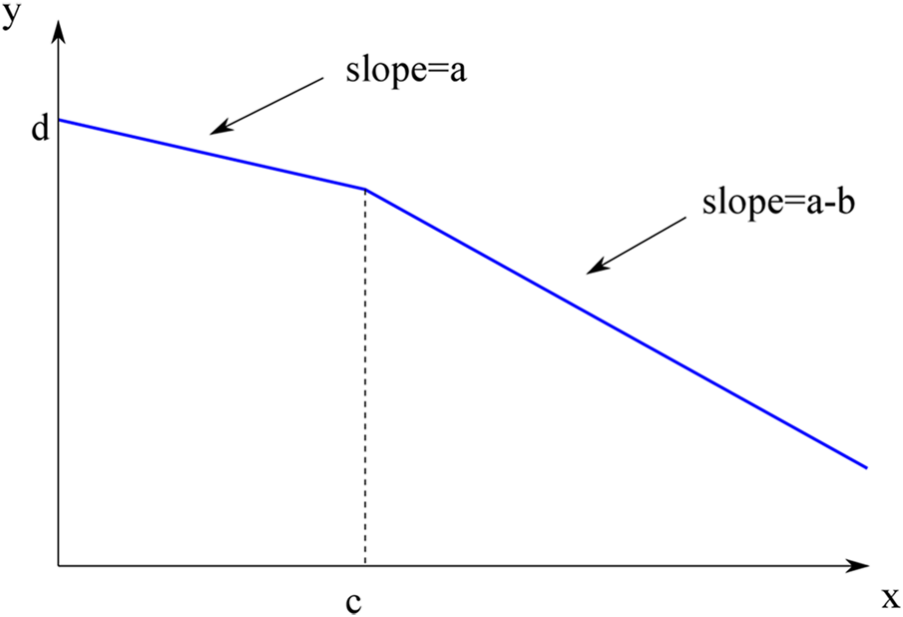
Graph of a two-segment piecewise function.

To predict the phase transition point, we considered the following function that provided a good empirical fit to our UV spectroscopy data,

1 $$y=\,\{\begin{array}{c}\,ax+d\\ ax-bx+bc+d\end{array}\begin{array}{c}\,\,\,\,\,if\,x < c,\\ \,\,\,\,otherwise\end{array}$$

where *y* represents AUC 240–250 nm (area under the absorption spectrum curve for UV light lengths 240–250 nm), *x* represents dilution index, and *a*, *b*, *c*, *d* are parameters to be fitted. This is a piecewise-linear function, with a different straight line segment for *x* < *c*, and a different one for *x* ≥ *c*, as shown in Fig. 5. Note that this function is everywhere continuous, including the point *x* = *c*.

The meaning of parameters is as follows: *a* is the slope of the first linear segment, *b* is the difference of slopes between the first and the second segment, *c* is the transition point between segments, and *d* is the intercept at *x* = 0. Note that when *b* = 0, the function reduces to a single straight line, and *c* becomes irrelevant in this case.

When the function given in eq. (1) was fit to experimental data by the method of least squares, using gnuplot program (www.gnuplot.info), it becomes clear that the most important parameter is *c*. If we can find *c* with high confidence, it means that the data indeed is well described by two-segment piecewise linear function. If, on the other hand, the fitting program has problems with finding *c* and reports very large uncertainty of *c*, this means that the eq. (1) is not a good model for the data.

We fitted eq. (1) to all 24 data sets (8 light honeys, 8 medium ones, and 8 dark ones). For dark honeys, the fit is very good, with values of *c* as shown in Table 3. The transition point in dilution is 2^C^, and this yields the values presented in Table 3, rounded to one digit after decimal point, for dark and medium honeys.

One can clearly see that the uncertainty in determining *c* reported by gnuplot is generally low, below 10%, with the exception of honey H125. For H220, the uncertainty is not given as gnuplot encountered singularity (cause to be determined). All phase transition points for dark honeys happens for dilutions between 8x to 32x and are in very good agreement with experimentally obtained phase transition points for these honeys (Table 3).

For medium honeys, the predicted phase transition point value *c* has higher uncertainty level, above 10%, clearly larger than in the case of dark honey. All phase transitions for medium honeys happened for dilutions between 4x and 16x, earlier than for dark honeys. Again, these data point agreed with that obtained experimentally (Table 3).

**Figure 6:**
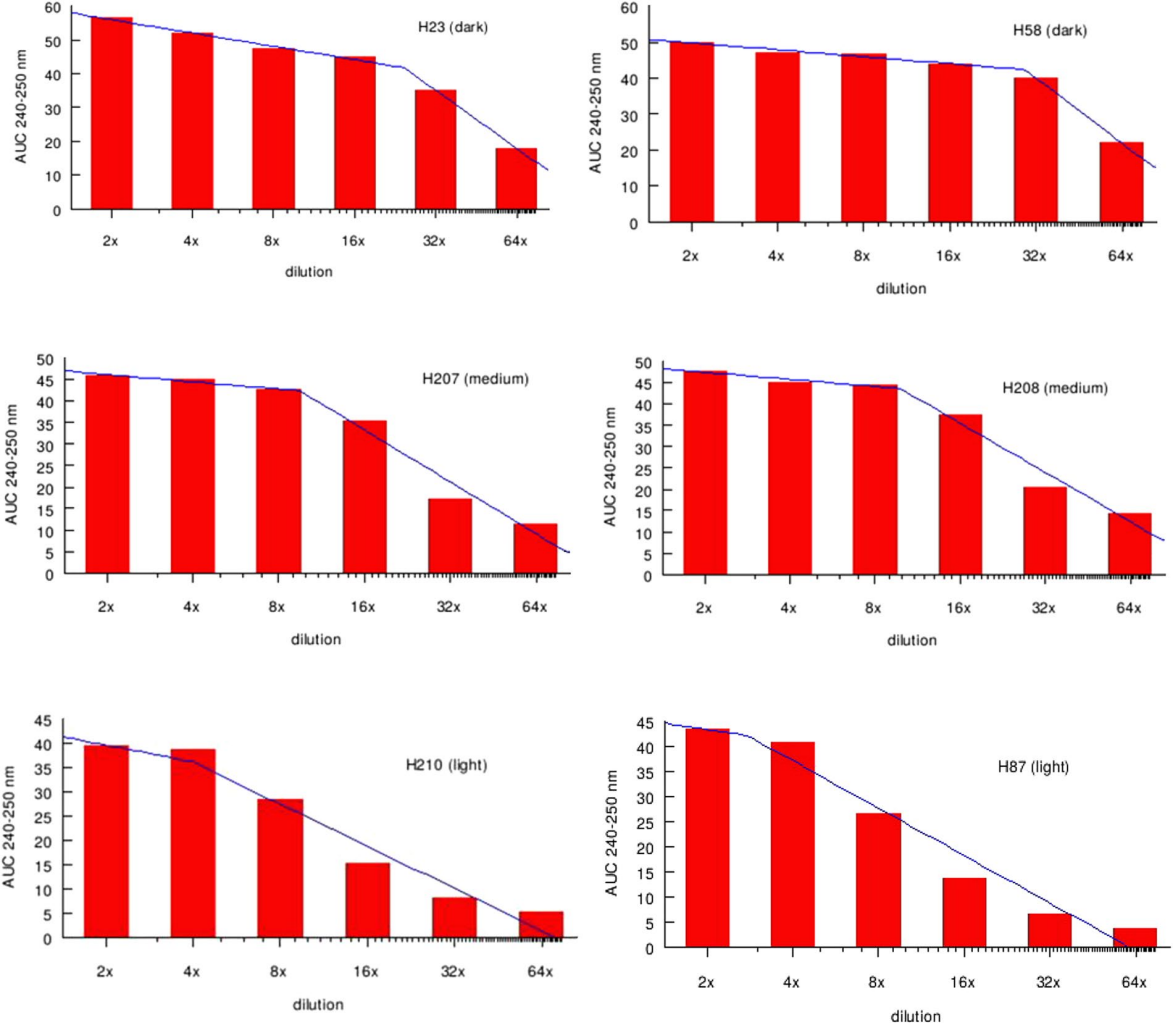
Typical fit for dark, medium and light honey to predict a phase transition point.

For light honeys, in almost all cases gnuplot reported huge and meaningless uncertainties in *c*. This is a sign that the fitting algorithm was not able to find a good fit, and, consequently, it means that eq. (1) is not a good model for the data for light honeys. The sole exception is H210, for which we obtained *c* =  2 ± 1.314 (65.68%). Figure 6 (bottom graph) shows a typical plot for the light honey, with failure of the fit clearly visible.

These results indicate that two-segment piecewise linear function is a good empirical model to explain the conformational changes in honey upon dilutions with respect to the concentration of UV absorbing compounds. The mathematical model supports the existence of two conformational states of honeys; one for honey with the high concentration of UV absorbing compounds and another under dilute solution conditions. The model predicts a phase transition point that separates these two conformations. The model failed to produce the phase transition point for light honeys indicating that their conformation is that of a dilute state conformation.

### Honey antibacterial activity depends on honey stable conformation and the phase transition point correlates with the MIC

We asked the question how the honey conformational changes affect its antibacterial activity. To examine this relationship, light, medium and dark honeys displaying the low to high values of AUC at the 200–400 nm and 240–250 nm were screened for their antibacterial activity against Gram-negative *E. coli* and *B. subtilis* using broth microdilution assay in the 96-well microplates (Table 4). The serial, two-fold dilution of honeys (from 2-fold to 64-fold dilution range) in this assay allowed determination of the Minimum Inhibitory Concentration (MIC) at which 90% of bacterial growth was inhibited.

**Table 3:** A comparison between the theoretical and experimental value of the phase transition point.

	Honey	Phase transition point (theoretical value) *c*	Dilution at Phase transition point 2^*c*^	Uncertainty level (%)	Dilution at phase transition (experimental value)
*Dark*	H76	3.379 ± 0.273	13.6	8.07	16x
	H226	3.7629 ± 0.3578	16.0	9.508	16x
	H58	3.99998 ± 0.3323	29.2	8.308	32x
	H77	4.8672 ± 0.07511	8.9	1.543	16x
	H23	3.15211 ± 0.145	24.5	4.6	16x
	H220	4.84809	29.9	na	32x
	H149	4.6148 ± 0.1121	12.6	2.429	16x
	H125	3.65302 ± 0.8858	10.4	24.25	8x
*Medium*	H96	2.50278 ± 0.6311	5.7	25.22	8x
	H207	3.2468 ± 0.5854	9.5	18.03	8x
	H99	2.71616 ± 1.05	6.6	38.65	8x
	H224	3.35079 ± 0.6782	10.2	20.24	8x
	H225	3.00433 ± 0.6788	8.0	22.59	8x
	H223	3.03744 ± 0.5916	8.2	19.48	8x
	H208	3.2912 ± 0.5425	9.8	16.48	8x
	H221	3.66029 ± 0.4262	12.6	na	8x

**Figure 7:**
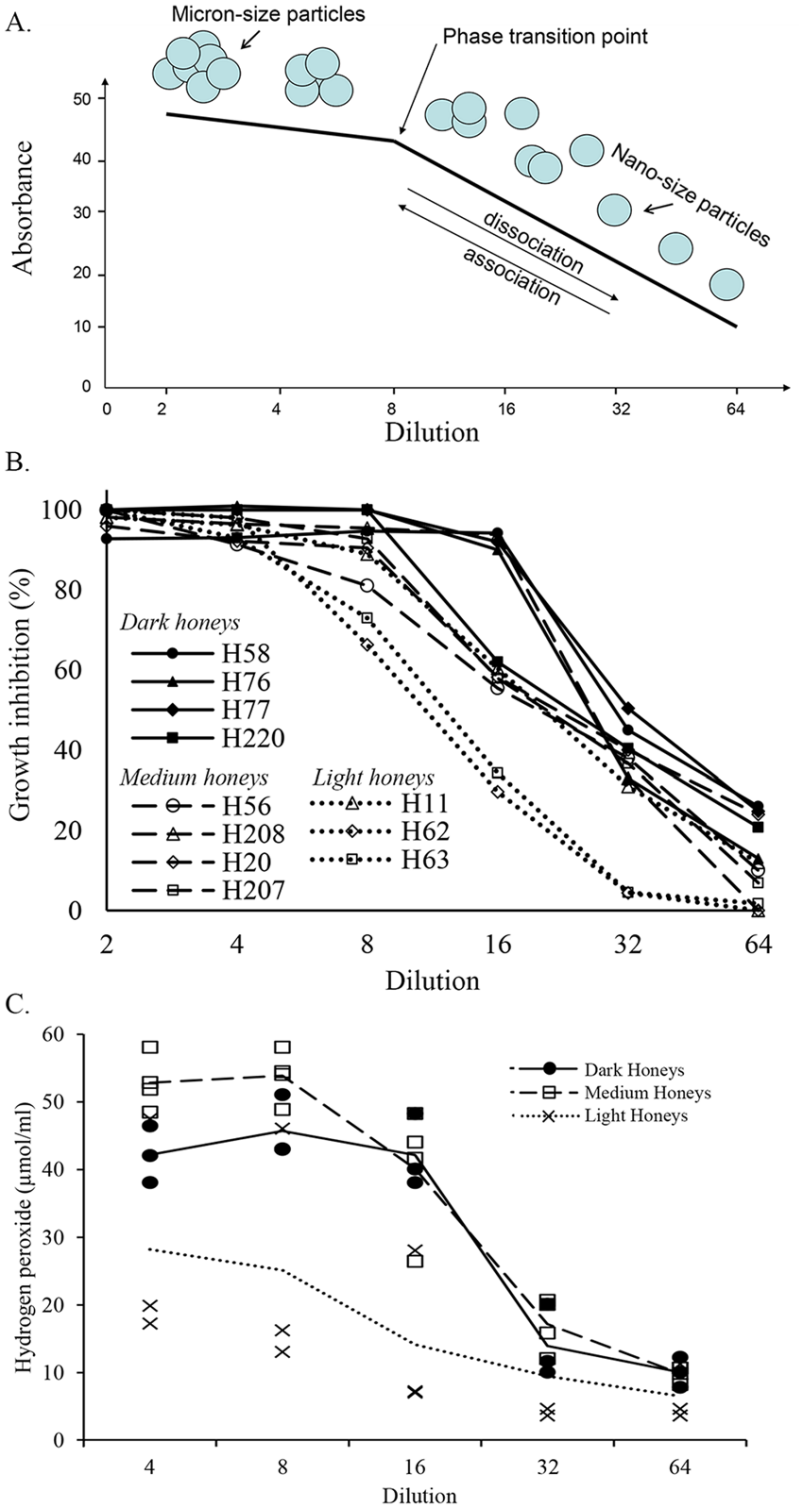
The relationship changes in honey structure upon dilution, a phase transition point and biological activities. (**A**) A schematic presentation of the assemblies of micron-size multimeric particles formed under molecular crowding and their dissociation, in orderly manner, to nano-size particles under dilute solution conditions. The phase transition point depicts the threshold concentration of >40 mAU at AUC 240–250 nm beyond which the multimeric particles undergo dissociation. The high polydispersidy index beyond the phase transition point indicates that the association and dissociation of particles is reversible. (**B**) The relationship between the growth inhibition and a phase transition point for dark, medium and light honeys. (**C**) The relationship between hydrogen peroxide generation and a phase transition point by dark, medium and light honeys.

Medium and dark color honeys containing high concentrations of UV absorbing compounds showed the highest antibacterial activity, with the MIC of 12.5 % to 6.25 % v/w corresponding to 8-fold and 16-fold dilution, respectively (Fig. 7B and Table 4). The light honeys with low to moderate AUCs values were much less active with the MIC ranging from 50% to 12.5% v/w corresponding to 2x to 8x dilution (Fig. 7B and Table 4).

**Table 4:** Relationship between honey antibacterial activity, AUC (240 nm–250 nm) and the phase transition point: Comparison of the AUC values at the corresponding honey dilution before and after the loss of the 240–250 nm peak.

	Honey	MIC		AUC (240–250 nm)	Phase transition dilution
		*E. coli*	*B. subtilis*		
Light Honeys	H210	8	4	38.7	4x
	H62	4	4	40.9	2x
	H87	4	4	40.9	4x
	H63	2	2	35.0	2x
	H68	4	4	41.6	2x
	H64	4	4	42.3	4x
	H11	8	4	42.7	4x
	H66	4	4	41.2	4x
Medium Honeys	H20	8	4	41.0	8x
	H96	8	8	43.9	8x
	H207	8	4	42.6	8x
	H99	8	4	43.9	8x
	H224	8	8	42.6	8x
	H225	8	8	41.7	8x
	H223	8	8	43.2	8x
	H208	8	8	44.5	8x
	H221	8	8	43.6	8x
Dark Honeys	H76	16	16	42.72	16x
	H226	8	8	42.04	16x
	H58	16	8	43.8	16x
	H77	16	8	45.78	16x
	H23	16	8	44.8	16x
	H220	8	8	45.27	16x
	H149	16	8	42.8	16x
	H125	8	8	45.8	8x

It became apparent that the difference in the concentration of UV absorbing compounds between medium/ dark honeys versus light honeys was a significant factor influencing antibacterial activity; t[23] = 10.046, p < 0.0001 for AUC 200–400 nm and t[23] = 5.163 p < 0.0001 for AUC 240–250nm, where the MIC average was 4.75 ± 0.75 for light honeys and 10.35 ± 0.91 for medium and dark honeys.

Next, we examine the relationship between honey conformation and its growth inhibitory activity taking the AUC value at 240–250 nm ≥40  mAU as the phase transition point. As shown above by DLS, SEM and mathematical model, the phase transition point separated two-phase system under macromolecular crowding from one-phase system under dilute solution conditions. Comparison of AUC 240–250 nm values of honeys with their MIC_90_ against *E. coli* revealed that all honeys with the concentrations of UV absorbing compounds above the phase transition point dilution (>40 mAU) inhibited growth of *E. coli* at 90%. The decrease of AUC values below 40 mAU resulted in a rapid loss of antibacterial activity ( Fig. 6). There was a significant correlation between honeys MIC_90_ against *E. coli* and *B. subtilis* and the phase transition point dilution; R = 0.5979, p < 0.0016 and R =  0.5132, p < 0.0246, respectively.

These results reveal a surprising and previously unknown fact that honey conformation under molecular crowding (AUC >40 mAU) was required in order to prevent bacterial growth.

### The active generation of hydrogen peroxide correlates with honey conformation under macromolecular crowding conditions and it ends at a phase transition point

Majority of North American and European honeys produce hydrogen peroxide as a by-product of glucose oxidation by glucose oxidase^17–19^. The levels of hydrogen peroxide parallel the antibacterial activity of honey and therefore H_2_O_2_ is considered a predictive biomarker of its antibacterial activity^19, 20^. To examine the relationship between honey conformation and hydrogen peroxide generation, we employed the sensitive AmplexRed method. In this method, honey H_2_O_2_ reacts stoichiometrically with 10-acetyl-3,7-dihydroxyphenoxazine (AmplexRed), oxidizing it into highly fluorescent resorufin in the HRP-catalyzed reaction. We quantified hydrogen peroxide generation at each dilution ranging from 4-fold to 32-fold in light, medium and dark honeys.

In medium and dark honeys, characterized by the high concentration of UV absorbing compounds (H77, H76 or H220, Fig. 7C, Table 5), the H_2_O_2_ levels increased with dilution, reaching the peak at 8-fold and 16-fold dilutions (corresponding to honey concentration of 12.5% and 6.25% v/w), respectively. After that, H_2_O_2_ levels sharply decreased (Fig. 7C). In contrast, in light honeys, a decrease in H_2_O_2_ production was observed throughout the entire dilution process (Fig. 7C).

**Table 5:** Summary of the relationship between the dilution at phase transition point, H_2_O_2_ production and MIC against *E. coli*.

	Honey	Phase transition point dilution	AUC 240–250 at phase transition point		H_2_O_2_ production at phase transition point		Dilution at max H_2_O_2_ production	MIC *E. coli*
			Before	After	Before	After		
Light Honeys	H210	2–4x	40	28.3	48	28	2–4x	8x
	H68	2x	41.6	26.8	14.4	13	2x	4x
	H62	2x	40.9	26.6	17.2	13	2x	2x
	H63	2x	35.4	20.7	19.8	16.2	2x	2x
	H87	4x	40.9	26.6	39.4	18	4x	4x
	H66	8x	41.4	27.0	50	36	8x	4x
	H11	4x	42.7	31.8	46	28	8x	8x
Medium Honeys	H20	8x	41.4	24.02	58	48	8x	8x
	H207	8x	42.6	35.4	54	44	8x	8x
	H208	8x	44.5	37.3	48.8	26	8x	8x
	H221	8x	43.6	37.9	54.4	40	4–8x	8x
	H99	8x	43.9	25.7	55.6	24	8x	8x
	H96	8x	43.9	26.8	54.2	38.6	8x	8x
Dark Honeys	H58	16x	40.0	21.9	46.6	36	8x	16x
	H77	16x	42.7	27	40	10	8x	16x
	H23	16x	44.8	35.1	39.8	18.8	4–8x	16x
	H220	16x	45.3	22.0	48.2	11.6	8–16x	8x
	H76	16x	42.7	27	38	20	8x	16x

A decrease in the production of H_2_O_2_ coincided with the phase transition point in medium and dark honeys. This observation was supported by the extremely significant quantitative difference in the H_2_O_2_ production before and after passing the phase transition point: R =  0.7124, p < 0.0009 (n = 18) (Table 5).

As in the case of antibacterial activity, honey conformation was the major factor underlying the H_2_O_2_ production. Honey two-phase conformation induced by macromolecular crowding supported hydrogen peroxide production, while honey conformation under dilute solution state (AUC 240–250 < 40mAU) did not (Table 5, Fig. 7C). Together, bacterial growth inhibition by honey and hydrogen peroxide production occurred only under molecular crowding conditions. Molecular crowding supported and maintained honey chemical reactivity demonstrating a direct link between honey global structure and its function.

## Discussion

Using an approach that combined UV spectral profiling, dynamic light scattering, SEM and mathematical model, we provided a novel view on global honey structure, its conformational phase transition with dilution and the effects of these structural changes on the antibacterial activity and hydrogen peroxide production.

Our hypothesis was that molecular crowding is decisive factor underlying global honey architecture and honey chemical reactivity against bacteria. Here, we present our most important findings that strongly relate to this new concept.

One of the key factors in determining honey structure was the concentration of honey macromolecules. This finding came from both UV spectroscopy and dynamic light scattering. UV spectroscopy provided a fast, qualitative and quantitative account on levels of UV absorbing compounds in different honeys based on the shape and absorbance intensity of UV spectra obtained from scans in the 200–400 nm range.

We quantified the UV absorbing compounds measuring the area under the curve (AUC) of UV spectral scans, knowing that the absorbance intensity was the sum of the absorbance and light scattering. We found that the concentration of UV absorbing compounds was the highest in dark > medium > light honeys and the differences among these groups were significant (p < 0.0001).

The concentration of UV absorbing compounds had a major impact on conformational stability of medium and dark honeys upon dilution. This could be explained by excluded volume effect. Higher concentrations of macromolecules exert larger excluded volume effect which in turn promotes stronger intermolecular interactions and formation of higher-order structures^4, 21, 22^. The formation of higher-order structures was supported by our results from dynamic light scattering and SEM. DLS results demonstrated that honey consisted of particles ranging from nano-size primary particles of 55 ± 5.2 nm to large, micron-size assemblies of 5.18 ± 0.38 μm. However, the percentage of micron-size particles was much higher in medium and dark honeys (35%) than in light honeys (5.6%) suggesting that the high concentration of macromolecules facilitated their self-assembly into large, super particles. SEM images gave a clear picture of large, dense, compact, spheroidal particles which were spatially distributed in sugar solution.

Our results are in good agreement with the theoretical and experimental data on the relationship between macromolecular concentration, excluded volume and intermolecular binding^4, 25^. In these studies, the non-ideal behavior of proteins under macromolecular crowding conditions was explained by a shift of the thermodynamic equilibrium of a macromolecular association reaction over dissociation rates in order to minimize the total free energy^4, 23–26^. Therefore, equilibrium theory^26^ predicted the formation of compact conformations via specific and nonspecific macromolecular associations and formation of oligomeric forms and aggregates, thereby supporting our results.

Our study indicated that the size and compactness of particles increased conformational stability against dilution. More water was required to weaken the association forces that kept the structure in compact form, to “unpack” them first and then to drive their dissolution. This increased resistance to water has been seen in our UV spectral profiling as a nonlinear decrease of the AUC values with the consecutive two–fold dilutions of medium and dark honeys, up to 16-fold dilution.

In contrast, destabilization of conformation occurred rapidly in light honeys. In light honeys, the low concentration of macromolecules resulted in formation of smaller size particles, with the average diameter ranging from 541.37 ± 73.13 nm and 259.85 ± 35.13 nm. The conformational integrity of small particles was readily compromised by dilution. In UV spectral profiling, it was seen as a linear decrease of the AUC values with the consecutive two–fold dilutions. Thus, the concentration of macromolecules was a crucial factor determining the size, compactness and stability of particles.

Dilution of honeys with water reduced the concentrations of crowding molecules and this in turn changed the properties of the solvent that allow unpacking and dissociating of large superstructures into smaller size particles. At the threshold concentration of molecular crowders, the conformational equilibrium was abruptly destabilized and the phase transition ensued. UV spectroscopy showed that the phase transition point had a measurable value of AUC 240–250 nm >40 mAU. Above the threshold concentration, a stable conformation was reflected by the elaborate UV spectra of high absorbance intensity that was likely caused by the high percentage of the large particles that both absorb and scatter the light as shown by DLS. Below the phase transition point (AUC 240–250 nm values  < 40 mAU), UV spectra of medium and dark honeys changed to simple, two-peak absorbance profiles. DLS results showed that dissociation of large, micro-size particles to smaller ones increased polydispersity index indicating the increase in the heterogeneity of particle sizes. DLS also showed that the particle fragmentation occurred in a quite orderly manner suggesting that the larger particles were formed from subunits that could self-assemble or disassemble. These results indicate that particle size is reversible depending on solvent conditions and macromolecule concentrations.

The phase transition point was a critical factor that separated two-phase system under macromolecular crowding, consisting of large, micron-size particles distributed in the highly concentrated sugar solution from one-phase system consisting of nano-size particles under dilute solution conditions. There was a direct link between honey global structure and its function. We observed that bacterial growth inhibition by honey and hydrogen peroxide production occurred only under molecular crowding conditions. Thus, molecular crowding supported and maintained honey chemical reactivity only if the concentration of macromolecules and crowding molecules, fructose and glucose was high enough to enforce reactants interactions.

Literature provides numerous examples of the increased enzymatic activity in several *in vitro* systems^24–26^. For example, it has been found and now commonly used in the laboratory that catalytic activity of T4 DNA ligase in blunt-end ligation is markedly enhanced in the presence of crowding molecules such as Ficoll, PEG 6000 and BSA^27, 28^.

Molecular crowding has been shown to substantially increase the hydrolysis of DNA by the endonucleases DNase I and S1 nuclease in the presence of polyethylene glycol (PEG)^29^. Somalinga and Roy^30^ showed that that crowding had a substantial effect on shift of equilibrium between protease-catalyzed peptide bond hydrolysis and its synthesis in reverse proteolysis if the difference between the volume excluded by the ligated product and the reactants was significantly large.

The picture that is gradually unfolding from our results is that honey is a colloidal system in which particles possess properties such as UV absorption and light scattering, and ability to form nano-size to micron-size hierarchical structures. The concentration of macromolecules determined the particle size and governed the particle optical properties.

Most importantly, two-phase colloidal system was required for honey antibacterial activity and hydrogen peroxide production. For the first time, a well-defined link had been established between the global honey structure under macromolecular crowding and its biological activities, such as antibacterial activity and hydrogen peroxide production.

Some indications as to chemical nature of macromolecules involved in particle formation came from our previous studies using size exclusion chromatography (SEC) and chemical characterization of peak fractions by LS/MS-MS, MALDI-TOF, and 1 and 2D SDS-PAGE^11, 12^. The SEC high molecular fractions were of multicomponent nature containing complexes of proteins, polyphenols and oligosugars. Among identified macromolecules were proteins belonging to Major Royal Jelly family^31^ and a broad range of phenolic acids and flavonoids that were involved in formation of protein-polyphenol complexes and melanoidins^32^. These macromolecules endow honey with antioxidant and antibacterial activities^31, 33, 34^.

The realization that honey active macromolecules are arranged into compact, stable multimeric assemblies reframes our view on honey global structure and emerges as a key property to be considered in investigating its biological activity.

## Methods

### Honeys

Honeys were donated by Canadian beekeepers and included both commercial and apiary samples. The samples included liquid and crystallized honeys and honeycomb honey from monofloral and polyfloral sources. Upon arrival to the laboratory, honeys were assigned the number and their basic physiochemical parameters were tested (Tables 1 and 2).

### Sample preparation

Honey samples were diluted to 50% (w/v) with warm (45–50 °C) Milli-Q water and the resulting solution was filtered using glass microfiber Centrex units (Schleicher & Schuell Inc. Keene, NH 03431, USA) followed by filtration through a 0.45 μm syringe membrane filter (VWR).

### Honey color

The mean absorbance of honey samples was determined using the method of Huidobro and Simal (1984)^35^ where the net absorbance of a 50% honey solution (w/v) was spectrophotometrically defined as the difference between the absorbance at 560 and 720 nm. Using these values, honeys were divided into three color-based groups (light, medium, dark) to facilitate the analysis of experimental data obtained in this study.

### Refractive index, °Brix and moisture content determination

Refractive index, Brix and moisture content were determined using an Abbe refractometer was performed according to the Harmonized methods of the European honey commission^36^. The instrument was calibrated at 20 °C and all measurements of honeys were performed at this temperature.

### Water activity

Honeys were serially diluted two-fold (1x–16x) with Mili-Q water. Water activity of the honey solutions was measured using an AquaLab Series 3 water activity meter (Decagon Devices, Inc., Pullman, WA). The instrument was calibrated with a salt solution (6.0 moles/kg NaCl, *a*_w_ = 0.760 ± 0.003) and Mili-Q water. Values reported represent the mean of two replicates.

### UV spectral analysis

Honeys were serially diluted from 2- to 64-fold with deionized Mili-Q water. An ultraviolet (200–400 nm) wavescan was performed for each solution using the Ultrospec 3100 pro UV/Vis spectrophotometer (Biochrom, Cambridge, UK) with deionized water as a blank. The integrated area under the curve (AUC) was computed in the ranges of 200–400 nm and 240–250 nm, using Swift II v2.03 software (Biochrom, Cambridge, UK).

### Dynamic Light Scattering

Dynamic light scattering experiments were carried out in a Zetasizer Nano-Zs (Malvern Instruments, Ltd., U.K.) using 173 degree backscattering angle. 5 runs of each sample were done with a minimum of 12 scans each. Backscatter system allows measuring smaller and weakly scatter samples with high sensitivity despite the presence of large particles. The honey samples were filtered through 0.45 μm filter before the measurements and serially, two-fold diluted with Mili-Q water.

### Scanning electron microscopy

Scanning electron microscopy images were obtained using an AMRAY 1600 Turbo SEM. Specimens were mounted onto a carbon adhesive tab and silver paint was applied to the specimen edges to aid in sample conductivity. Using a secondary electron scintillation detector and 15 kV accelerating voltage, images were processed using ORION digital imagegrabbing software ver. 6.51. Calibrated lm-scaled bars are incorporated in the CRT output of the AMRAY SEM.

### Antibacterial susceptibility assay

Standard strains of *Bacillus subtilis* (ATCC6633) and *Escherichia coli* (ATCC 14948) purchased from Thermo Fisher Scientific Remel Products (Lenexa, KS 66215, USA) were grown in Mueller–Hinton Broth (MHB; Difco Laboratories) overnight in a shaking water- bath at 35 °C. Overnight cultures were diluted with broth to the equivalent of the 0.5 McFarland Standard.

The antibacterial activity of honeys was performed using a broth microdilution assay in sterile, 96-well microplates (Costar, Thermo Fisher Scientific, Canada) in compliance with requirements of CLSI (2015). Wells were filled with serially two-fold diluted honeys in Mueller-Hinton broth (100 μl) and inoculated with 10 ul of 10^6^ CFU/ml either *E. coli* or *B. subtilis*. A positive control (bacterial inoculum) and negative control (uninoculated broth) were included in the test. Honeys were tested in triplicate. Plates were incubated at 35 °C in a shaking water-bath, for 18 h. Bacterial growth was measured at A595 nm using the Synergy HT microplate reader (Bio-tek, Instruments,Winooski, VT, USA). Statistical analysis and dose–response curves were obtained using K4 software provided by Synergy HT, Bio-Tek Instruments, Winooski, VT, USA. The MIC endpoint was the lowest concentration of honey that completely inhibited growth of the bacteria.

### Hydrogen peroxide measurements

The concentration of hydrogen peroxide in honeys was determined using the Amplex Red Hydrogen Peroxide/Peroxidase Assay Kit in 96-wellmicroplate format according to the manufacturer manual (Molecular Probes, Invitrogen, Burlington, ON, Canada) and as described previously^20^. The Synergy HT multidetection microplate reader was used to measure the fluorescence formed during the reaction of honey’s hydrogen peroxide with the AmplexRed reagent (10-acetyl-3,7 dihydroxyphenoxasine). The measurements were conducted at an emission wavelength of 590 nm and an excitation wavelength of 530 nm. The standard curve constructed from the known concentration of H_2_O_2_ was used to calculate the hydrogen peroxide concentrations of the honeys. Each of the honeys samples, and the standard curve were tested in triplicate.

### Statistical analysis

Analyses were performed using the statistical program Graph-Pad Instat version 3.05 (GraphPad Software Inc.). Data were analysed using a one-way ANOVA with subsequent Tukey–Kramer Multiple Comparison test or an unpaired t-test. Differences between means were considered to be significant at p < 0.05.

### Data availability

all data underlying the findings described in their manuscript are fully available.
